# High-Dose *Mycobacterium tuberculosis* H37rv Infection in IL-17A- and IL-17A/F-Deficient Mice

**DOI:** 10.3390/cells11182875

**Published:** 2022-09-14

**Authors:** Kristina Ritter, Jochen Behrends, Dominik Rückerl, Alexandra Hölscher, Johanna Volz, Immo Prinz, Christoph Hölscher

**Affiliations:** 1 Infection Immunology, Research Center Borstel, D-23845 Borstel, Germany; 2 Fluorescence Cytometry Core Unit, Research Center Borstel, D-23845 Borstel, Germany; 3 Lydia Becker Institute of Immunology and Inflammation, School of Biological Sciences, University of Manchester, Manchester M13 9PL, UK; 4 Center for Molecular Neurobiology Hamburg, Eppendorf University Medical Center, D-20246 Hamburg, Germany; 5 Thematic Translational Unit Tuberculosis, German Center for Infection Research (DZIF), D-38124 Braunschweig, Germany

**Keywords:** tuberculosis, rodent, cytokines, IL-17A, IL-17F, neutrophils

## Abstract

During experimental tuberculosis (TB), interleukin (IL)-17A appears to be involved in the formation of lung granulomas, possibly through the attraction of neutrophils to the sites of infection. However, the protective impact of cytokine appears to depend on the degree of its induction. Hence, robust production of IL-17A in mice infected with the hypervirulent isolate *Mycobacterium tuberculosis* (Mtb) HN878 mediates protection, while the cytokine is dispensable for protective immune responses against low-dose infection with the less virulent strain H37rv. Here, we show that after experimental infection with high doses of Mtb H37rv, IL-17A-deficient (^−/−^) mice exhibited high susceptibility to the infection, which was mediated by the strong accumulation of neutrophils in the infected lung tissue. Accordingly, we observed nearly unrestricted bacterial replication within the neutrophils, indicating that they may serve as a survival niche for Mtb. By use of IL-17A/IL-17F-double-deficient mice, we demonstrated that the susceptibility in the absence of IL-17A is mediated by a compensatory expression of IL-17F, which, however, appeared not to be dependent on neutrophils. Together, our results illustrate the compensatory potential of the Th17-secreted cytokines IL-17A and IL-17F in the context of experimental TB and once again emphasize the detrimental effect of excessive neutrophil infiltration in response to Mtb.

## 1. Introduction

After COVID-19, tuberculosis (TB) is still the deadliest infectious disease worldwide [1]. In 2020, approximately 10 million people fell ill with active TB and 1.5 million patients died from the disease. A profound understanding of the cellular components and protective immune mechanisms during infection with *Mycobacterium tuberculosis* (Mtb) is mandatory for the successful development of novel drugs and effective vaccines against TB.

It is widely accepted that CD4^+^ T helper 1 (Th1) cells, which express the cytokines interferon-gamma (IFN-γ) and tumor-necrosis-factor (TNF), play a major role during infection with Mtb [2,3,4]. Both these cytokines are able to induce anti-mycobacterial effector mechanisms in the Mtb-infected macrophages [5]. In addition to IFN-γ and TNF, the pro-inflammatory cytokine IL-17A also appears to contribute to protective immune responses against infection with Mtb [6,7,8]. IL-17A represents the signature cytokine of another CD4^+^ T cell subset, Th17 cells. In the context of experimental TB, the expression of these Th17 cells is mediated by IL-23 [9,10], but is largely independent of IL-6 [11]. Besides Th17 cells, IL-17A is also expressed by certain innate cell types such as gamma-delta (γδ) T cells [12]. The cytokine is upregulated during TB in mice [9] and humans [13]. After experimental infection with *Mycobacterium bovis* bacille Calmette-Guérin (BCG) [14,15] as well as Mtb [6,7], IL-17A contributed to the formation and maturation of lung granulomas. It appears to be involved in the instruction of Th1 immune responses but it also promotes the recruitment of neutrophils in the infected lung [15]. However, the protective impact of IL-17A during experimental TB seems to be dependent on the degree of its induction. Accordingly, it is not required for the development of protective immune responses against infection with either the laboratory-adapted Mtb strain H37rv or the less virulent Mtb clinical isolate CDC1551 [7]. In contrast, after infection with the hypervirulent Mtb strain HN878, IL-17A mediates early protection through induction of the C-X-C motif chemokine CXCL13, which, in turn, supports the localization of C-X-C chemokine receptor type (CXCR)5-expressing T cells within lung lymphoid follicles to ensure optimal macrophage activation and thus control of Mtb infection. Infection with HN878 thus leads to a potent induction of IL-17A [7], suggesting that the extent of IL-17A-mediated anti-mycobacterial protection correlates with an overproduction of the cytokine. Thus, in response to certain vaccine candidates against TB, the early induction of IL-17A conveys vaccine-induced protection from Mtb challenge, but in vaccination with other candidates, the cytokine is dispensable [8,16]. Similarly, the increased production of IL-17A in mice with a deficiency of the specific IL-27 receptor subunit IL-27Rα mediated protection against Mtb accompanied by the formation of highly organized granulomas and an accumulation of multifunctional CD4^+^ T cells in the lung [6,17].

On the basis of these findings, we speculated that, in contrast to the minor impact of IL-17A during a low-dose infection with Mtb H37rv, the cytokine may exert a larger influence in response to infection with higher doses of the laboratory-adapted Mtb strain. To prove this hypothesis, in the present study, we analyzed the outcomes of experimental TB in IL-17A-deficient (^−/−^) mice infected via an aerosol with different doses of Mtb H37rv. Indeed, the relevance of IL-17A in the induction of protective immune responses against Mtb appeared to correlate with the infectious dose. Thus, after a high-dose Mtb infection, IL-17A^−/−^ mice exhibited significantly elevated bacterial burdens, surprisingly accompanied by a massive infiltration of neutrophils into the infected lung alongside elevated gene expression of *Il17f*. Lastly, by use of mice with a simultaneous deficiency in the closely related cytokines IL-17A and IL-17F, we identified IL-17F as mediating the increased susceptibility in the absence of IL-17A after high-dose infection with Mtb H37rv, while it appeared to exert a minor impact on the enhanced infiltration of neutrophils in the IL-17A^−/−^ mice.

## 2. Materials and Methods

### 2.1. Mice

Breeding pairs of IL-17A^−/−^ (MGI nomenclature: B6.129P2-*Il17a*^tm1Yiw^) [18] and IL-17A/F^−/−^ mice (MGI nomenclature: B6.Cg-*Il17a/Il17f*^tm1.1Impr^ Thy1a) [19] were kindly provided by Yoichiro Iwakura (University of Tokyo, Tokyo, Japan) and the Hannover Medical School (Hannover, Germany), respectively. Both strains have a C57BL/6 genetic background. The animals were bred at the Institute of Animal Breeding and Husbandry at the Christian-Albrechts-University (Kiel, Germany). Wildtype C57BL/6 mice were obtained from Charles River (Sulzfeld, Germany). In all experiments, female mice were used exclusively. During the experiments, animals were maintained in individually ventilated cages in the BSL 3 facility at the Research Center Borstel (Borstel, Germany). All procedures including experimental animals were executed in agreement with the German animal protection laws and were approved by the local authorities at the Ministry of Energy, Agriculture, the Environment, Nature, and Digitalization (Kiel, Germany) (approval numbers 83-7/06 and 28-3/10).

### 2.2. Bacteria and Infection

In all experiments, mice were infected with either <100 colony-forming-units (CFU), 100–1000 CFU, or >1000 CFU per lung of the lab-adapted Mtb strain H37rv using an inhalation exposure system (Glas-Col, Terre-Haute, IN, USA) as described [16]. The following day, the actual dose of infection was verified by determining CFUs in the entire lung of representative mice. Signs of severe stress were defined as a humane endpoint, where the infected animals were euthanized without pain.

### 2.3. In Vivo Depletion of Neutrophils

To deplete neutrophils in vivo, animals received intravenous injections of 100 µL of an anti-granulocyte-colony-stimulating factor (anti-G-CSF) sheep antiserum intravenously on Days -5 and -3 of infection [20] (kindly provided by Corinna Herrmann, University of Konstanz, Konstanz, Germany). The degree of neutrophil depletion was eventually determined in blood smears as described in [20].

### 2.4. Colony Enumeration Assay

Bacterial burdens in the lungs of experimental animals were determined at different time points of infection as described in [16].

### 2.5. Histology

Sections measuring 2 µm were prepared from the formalin-fixed and paraffin-embedded left lungs of infected mice. For histopathological examinations and detection of acid-fast bacilli in lung sections, standard protocols for hematoxylin/eosin (H&E) and Ziehl–Neelsen staining were used, respectively [16]. Quantification of the proportions of inflammatory cellular infiltrates within the total area of 4–8 serial sections of the lung was performed using ImageJ 1.53e (National Institute of Mental Health, Bethesda, MD, USA) as described in [21]. For each of the serial sections, the same color threshold parameters were used as a basis for digital measurements. To detect NOS2 or neutrophils by immunohistochemistry, antigen-retrieval in lung sections were performed as previously described in [22]. After peroxidase quenching and blocking [16], samples were incubated with a rabbit-anti-NOS2 antibody (Merck Millipore, Burlington, MA, USA) or a rat-anti-neutrophil [7/4] antibody (Cedarlane Laboratories, Burlington, ON, Canada) overnight. After incubation with a biotin-labelled goat-anti-rabbit secondary antibody (Dianova, Hamburg, Germany) or a biotin-labelled rabbit anti-rat antibody (Vector Laboratories, Newark, CA, USA), respectively, color development was performed as described in [16]. Quantification of the proportions of neutrophil infiltrates within the total area of 4–8 serial sections of lung was performed by means of a geometric grid. 

### 2.6. Preparation of Single-Cell Suspensions from Infected Lungs

At different time points, single-cell suspensions from the lungs of infected animals were prepared. Lungs were perfused by injecting warm PBS through the right ventricle. After digestion of coarsely shredded lungs in Collagenase A (0.7 mg/mL, Roche Diagnostics, Mannheim, Germany) and DNase (30 µg/mL, Sigma-Aldrich, St. Louis, MO, USA) at 37 °C for 2 h, the lung tissue was dislocated by pressing through a nylon cell strainer with a 100 µm pore size. After depletion of the remaining erythrocytes using a hypotonic red cell lysis buffer, lung cell suspensions were resuspended in complete Iscove’s modified Dulbecco’s medium (IMDM; PAA Laboratories GmbH, Cölbe, Germany) supplemented with 10% FCS, 1% L-glutamine (200 mM; Biochrom, Berlin, Germany), and 1% penicillin/streptomycin (10,000 U/mL and 10,000 mg/mL; Biochrom).

### 2.7. Flow Cytometry

To determine the absolute numbers of Gr-1^+^ CD11b^+^ granulocytes in the lungs from infected animals, flow cytometric analysis of single-cell suspensions was performed. After blocking of nonspecific binding by adding an anti-FcγRIII/II antibody (BioLegend, Amsterdam, The Netherlands) and a cocktail of mouse, rat, and hamster serum, cells were incubated with optimal amounts of the following specific antibodies (all BD Biosciences, Heidelberg, Germany): anti-CD8-V450, anti-CD4-V500, anti-CD11c-FITC, anti-CD11b-PE, anti-Ly-6G-PerCP-Cy5.5, anti-CD90.2-PE-Cy7, anti-Gr-1-APC, and anti-MHCII(IA/IE)-APC-e780. Measurement was performed on a FACSCanto™ II (BD Bioscience) and the FCS files were analyzed using the FCS Express 7 Flow Cytometry software (DeNovo™ Software, Pasadena, CA, USA). Absolute cell numbers were calculated as follows: (total cell count lung/100) × % of Gr-1+ CD11b+ cells (of analyzed flow cytometric leukocytes) = absolute cell numbers.

### 2.8. Quantitative Real-Time PCR

Lung tissue was homogenized in a 4 M guanidinium–isothiocyanate buffer. RNA extraction, reverse transcription into cDNA, and quantitative PCR conducted using a Light Cycler^®^ 480 Instrument (Roche Diagnostics) were performed as described in [23]. Data were analyzed by the second derivative maximum method and the standard curve method using hypoxanthine–guanine phosphoribosyltransferase (*Hprt*) as a housekeeping gene to calculate the level of gene expression in relation to *Hprt*. The following primer and probe sets (Roche Diagnostics) were used: *Hprt*: sense 5′-TCC TCC TCA GAC CGC TTT T-3′, antisense 5′-CCT GGT TCA TCA TCG CTA ATC-3′, probe #95′; *Il22*: sense 5′-TTT CCT GAC CAA ACT CAG CA-3′, antisense 5′-TCT GGA TGT TCT GGT CGT CA-3′, probe #17; *Il17f*: sense 5′-CCC AGG AAG ACA TAC TTA GAA GAA A-3′, antisense 5′-CAA CAG TAG CAA AGA CTT GAC CA-3′, probe #46; *Il12p40*: sense 5′-ATC GTT TTG CTG GTG TCT CC -3′, antisense 5′-GGA GTC CAG TCC ACC TCT ACA-3′, probe #78; and *Il21*: sense 5′-GAC ATT CAT CAT TGA CCT CGT G-3′, antisense 5′-TCA CAG GAA GGG CAT TTA GC-3′ probe #27.

In the case of *Il23p19*, PCR was conducted using the Light-Cycler-DNA Master SYBR Green^®^ I kit (Roche Diagnostics) and the following primer sets:

*Hprt*: sense *5′-GCA GTA CAG CCC CAA AAT-3′*, antisense 5′-AAC AAA GTC TGG CCT GTA TCC AA-3′; *Il23p19*: sense 5′-TCC CTA CTA GGA CTC AGC CAA C-3′, antisense 5′-TGG GCA TCT GTT GGG TCT-3′.

### 2.9. Statistics

Data are expressed as the means of individual determinations and the standard deviations. Statistical analysis was performed using Prism 9 (GraphPad Software, San Diego, CA, USA). The Kruskal–Wallis test with Dunn’s multiple comparison test or, if applicable, a two-way ANOVA with a Bonferroni post hoc test was applied. Statistical analysis of the survival of infected mice was conducted using the log-rank test. For *p* values ≤ 0.05, the results were considered statistically significant.

## 3. Results

### 3.1. The Requirement of IL-17A for Controlling Bacterial Growth Depends on the Dose of Infection with Mtb H37rv

**Figure 1:**
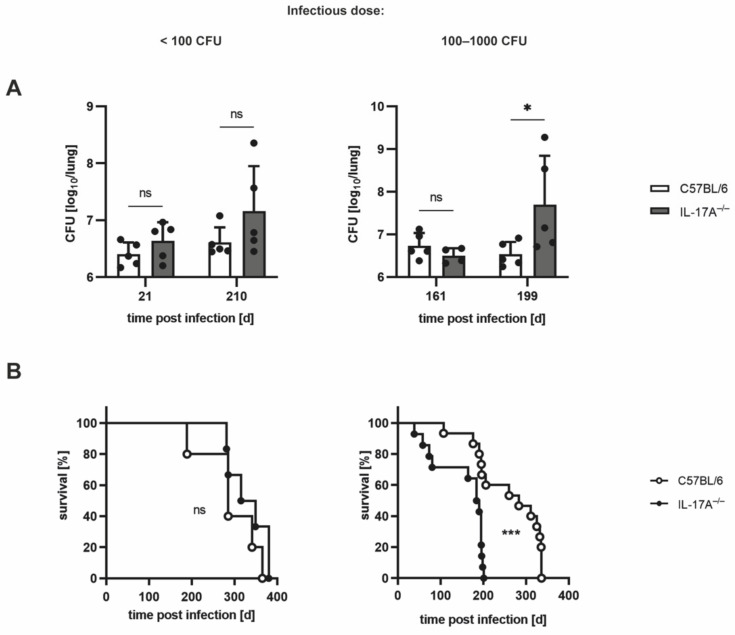
After infection with elevated doses of Mtb, IL-17A^−/−^ mice exhibit impaired control of mycobacterial growth and accelerated death. (**A**,**B**) C57BL/6 and IL-17A^−/−^ mice were infected with <100 CFU (left) or 100–1000 CFU (right) of H37rv Mtb by an aerosol. (**A**) At different time points of infection, CFUs in the lungs were determined. Data are presented as single dots and as the mean ± SD of 4–5 mice per group from one representative experiment (100–1000 CFU) or from one representative experiment out of two performed (<100 CFU). Statistics were calculated for log-transformed data using a two-way ANOVA with Bonferroni’s post hoc test. Differences between C57BL/6 and IL-17A^−/−^ mice were defined as significant (ns *p* ≥ 0.05; * *p* < 0.05). (**B**) The survival of 5–15 mice per group was recorded using humane endpoints. Statistical analysis was accomplished using the log-rank test. Differences in the survival kinetics between C57BL/6 and IL-17A^−/−^ mice were defined as significant (ns *p* ≥ 0.05; *** *p* < 0.001).

Since it has been demonstrated that the requirement of IL-17A for protective immune responses to Mtb depends on the virulence of the Mtb strain used for infection [7], we speculated that IL-17A may similarly mediate protection after infection of the experimental mice with an elevated dose of Mtb H37rv. Accordingly, we compared the outcome of Mtb infection in C57BL/6 and IL-17A^−/−^ mice infected with <100 CFU (low dose) with mice infected with an elevated infectious dose of 100–1000 CFU of Mtb H37rv (Figure 1). After infection with <100 CFU, the mycobacterial burdens in IL-17A^−/−^ mice were slightly but not significantly higher than those in C57BL/6 wildtype animals (Figure 1A; left), corroborating previous findings [7,16] showing that IL-17A contributes to containment of a low-dose Mtb infection to only a minor degree. When infected with an elevated dose of Mtb, however, IL-17A^−/−^ mice exhibited significantly enhanced bacterial loads at later but not at earlier time points of infection (Figure 1A; right).

Similarly, monitoring the survival of C57BL/6 and IL-17A^−/−^ mice infected with different doses of Mtb H37rv also revealed differential requirements for IL-17A (Figure 1B). Whereas after infection with <100 CFU Mtb, wildtype and IL-17A^−/−^ mice had to be euthanized with a similar kinetic, infection with an elevated dose of Mtb resulted in a significantly diminished survival time in the mutant mice (Figure 1B). In the latter case, the median overall survival time was 283 days for C57BL/6 mice, while IL-17A^−/−^ mice had to be euthanized approximately 100 days earlier, with a median overall survival time of 187 days (Figure 1B; right).

**Figure 2:**
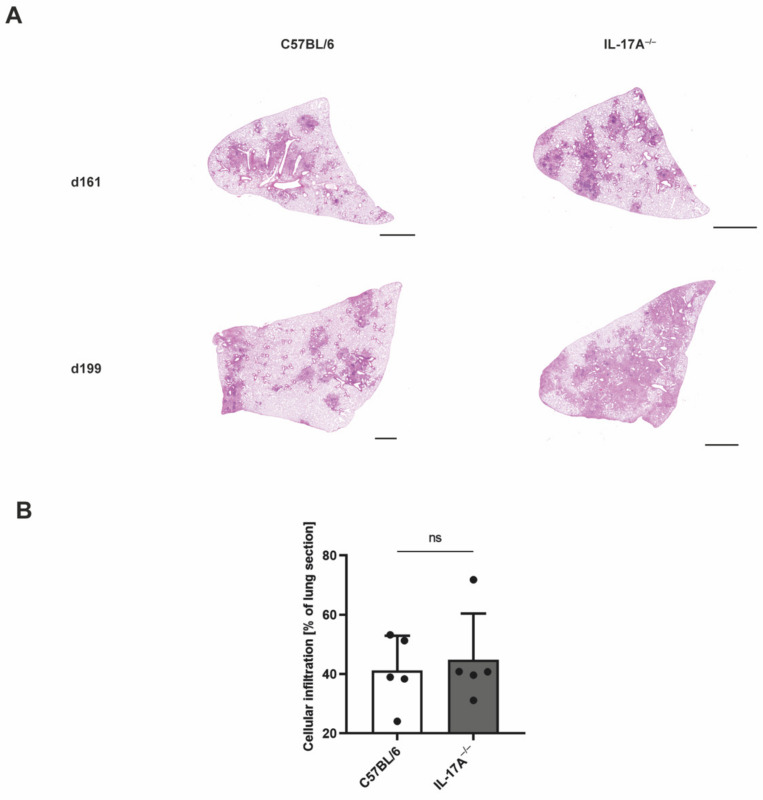
After infection with an elevated dose of Mtb, the lungs of IL-17A^−/−^ mice show similar inflammatory cellular infiltrates. C57BL/6 and IL-17A^−/−^ mice were infected with 100–1000 CFU of H37rv Mtb by an aerosol. Formalin-fixed lung sections taken on Days 161 and 199 post-infection were stained with H&E. (**A**) Representative lung sections from 1 out of 5 mice per group are shown (magnification: 40×, bar: 2 mm). (**B**) Proportions of cellular infiltration within the total area of the lung sections taken on Day 199 post-infection were determined in 4–8 serial sections per animal. Data are presented as single dots and as the mean ± SD of 5 mice per group from one representative experiment. Statistical analyses were conducted using a Mann-Whitney test. Differences in the survival kinetics between C57BL/6 and IL-17A^−/−^ mice were defined as significant (ns *p* ≥ 0.05).

To check whether, in accordance with the increased susceptibility of IL-17A^−/−^ mice to infection with elevated doses of Mtb H37rv, the inflammatory cellular infiltration in IL-17A^−/−^ mice was also augmented, and the lung sections of the animals were histologically analyzed (Figure 2). The proportions of cellular infiltration within the total area of the lung sections, however, were rather similar in the wildtype and IL-17A^−/−^ mice infected with a higher dose of Mtb (Figure 2A,B).

### 3.2. Mtb-Containing Lesions in IL-17A^−/−^ Mice after Infection with an Elevated Dose of Mtb Are Partly Associated with Enhanced Numbers of Neutrophils

**Figure 3:**
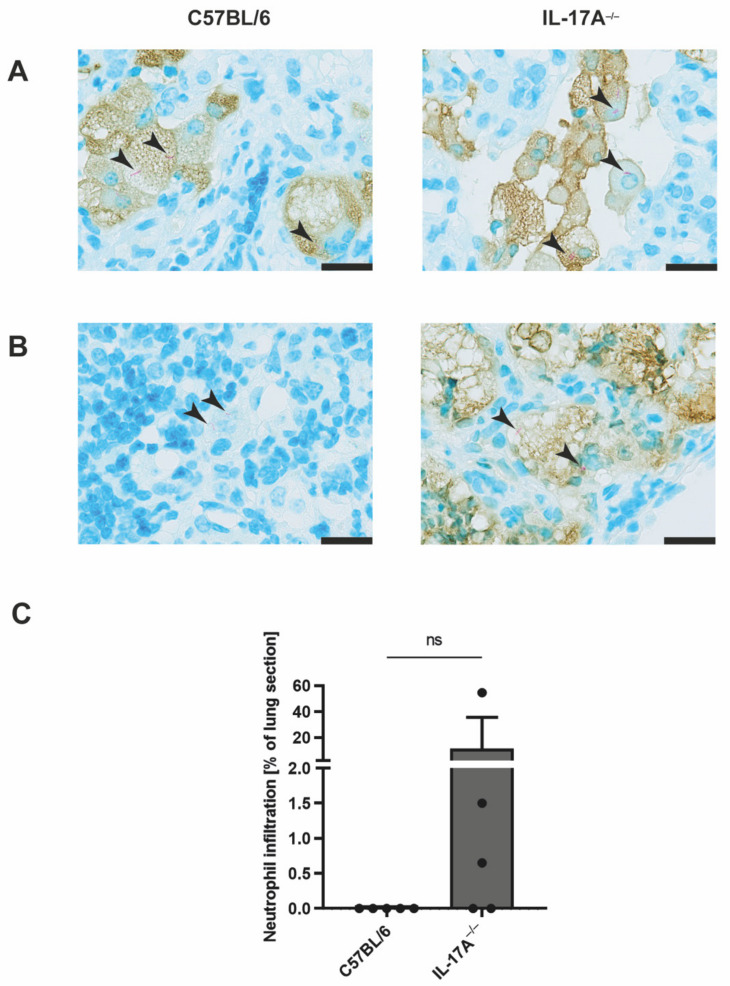
In the absence of IL-17A, infection with elevated doses of Mtb partially provokes increasing infiltration of Mtb-containing neutrophils. (**A**–**C**) C57BL/6 and IL-17A^−/−^ mice were infected with 100–1000 CFU of H37rv Mtb by an aerosol. In formalin-fixed lung sections taken on Day 199 post-infection, acid-fast bacilli were detected by Ziehl–Neelsen staining. In addition, the sections were co-stained for NOS2 (**A**) or neutrophils (**B**,**C**). (**A**,**B**) Representative sections from 1 out of 5 mice per group are shown (magnification: 1000×, bar: 20 µm, black arrows: acid-fast bacilli). (**C**) Proportions of neutrophil infiltration within the total area of the lung sections were determined in 4–8 serial sections per animal. Data are presented as single dots and as the mean ± SD of 5 mice per group from one representative experiment. Statistical analyses were conducted using a Mann-Whitney test. Differences in the survival kinetics between C57BL/6 and IL-17A^−/−^ mice were defined as significant (ns *p* ≥ 0.05).

To further assess the cellular composition of the Mtb-containing lesions in IL-17A^−/−^ mice, we performed immunohistochemical evaluation of lung sections from C57BL/6 and IL-17A^−/−^ mice after infection with an elevated dose of Mtb H37rv (Figure 3). Co-staining for acid-fast bacilli and the macrophage effector molecule NOS2 showed similar proportions of Mtb-infected activated macrophages in wildtype and mutant mice (Figure 3A). In contrast, staining for acid-fast bacilli along with an anti-neutrophil [7/4] antibody revealed that IL-17A^−/−^ mice partly exhibited an increased infiltration of Mtb-containing neutrophils into the lungs when compared with the C57BL/6 controls (Figure 3B). However, due to heterogenicity within the group of IL-17A^−/−^ mice, a quantitative analysis of the neutrophil infiltration did not reveal a significant difference between the experimental groups (Figure 3C).

### 3.3. The Increased Infiltration of Neutrophils Is Involved in the Strongly Enhanced Susceptibility of IL-17A^−/−^ Mice after Infection with a High Dose of Mtb H37rv

**Figure 4:**
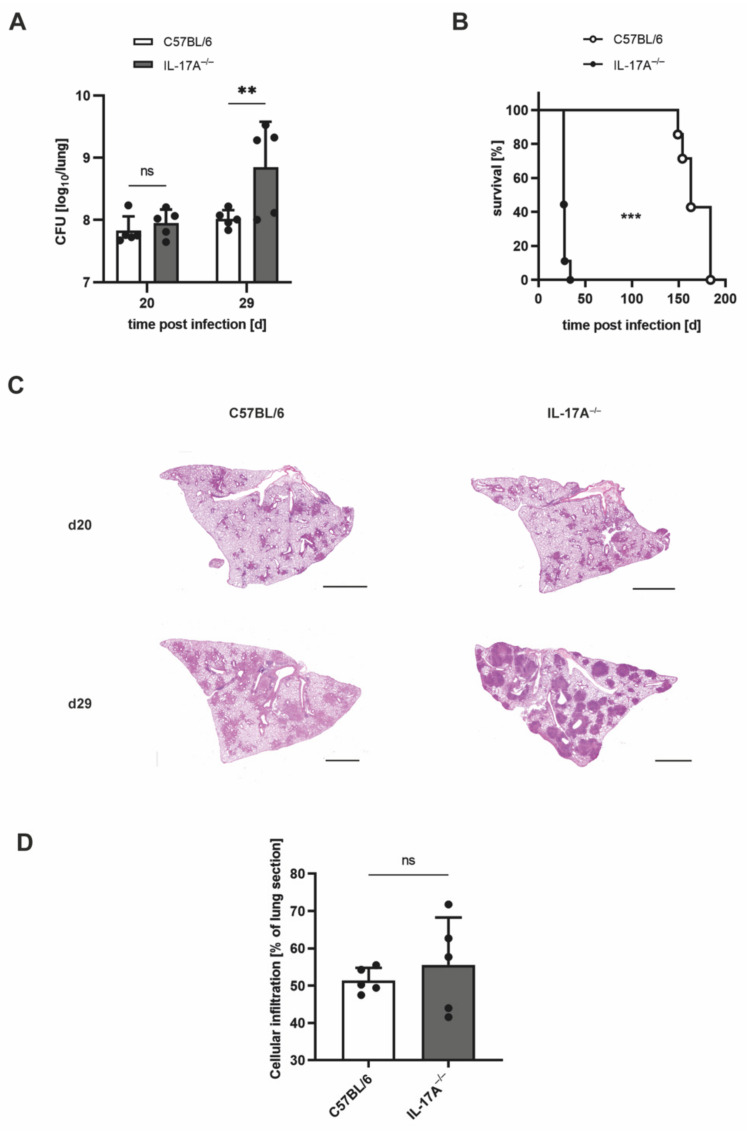
After ultrahigh-dose infection with Mtb, considerably shortened survival in IL-17A^−/−^ mice is partially accompanied by pronounced granulomatous lesions in the lung. (**A**–**D**) C57BL/6 and IL-17A^−/−^ mice were infected with >1000 CFU of H37rv Mtb by an aerosol. (**A**) At different time points of infection, CFUs in the lungs were determined. Data are presented as single dots and as the mean ± SD of 5 mice per group from one representative experiment. Statistics were determined for log-transformed data using a two-way ANOVA with Bonferroni’s post hoc test. Differences between C57BL/6 and IL-17A^−/−^ mice were defined as significant (ns *p* ≥ 0.05; ** *p* < 0.01). (**B**) The survival of 7–9 infected mice per group was recorded using humane endpoints. Statistical analyses of the resulting survival curve were accomplished using the log-rank test. Differences in the survival kinetics between C57BL/6 and IL-17A^−/−^ mice were defined as significant (*** *p* < 0.001). (**C**,**D**) Formalin-fixed lung sections taken on Days 20 and 29 post-infection were stained with H&E. (**C**) Representative lung sections from 1 out of 5 mice per group are shown (magnification: 40×, bar: 2 mm). (**D**) Proportions of cellular infiltration within the total area of the lung sections taken on Day 29 post-infection were determined in 4–8 serial sections per animal. Data are presented as single dots and as the mean ± SD of 5 mice per group from one representative experiment. Statistical analyses were conducted using a Mann-Whitney test. Differences in the survival kinetics between C57BL/6 and IL-17A^−/−^ mice were defined as significant (ns *p* ≥ 0.05).

On the basis of the association of the enhanced influx of neutrophils and the increased bacterial loads in the absence of IL-17A, we speculated that neutrophils may mediate the susceptibility of IL-17A^−/−^ mice to Mtb. To prove this assumption, we aimed to deplete neutrophils in vivo with an anti-G-CSF anti-serum [20]. It was demonstrated earlier that by an injection of this serum, in vivo depletion of neutrophils occurred with a similar efficiency as achieved with an anti-Ly-6G antibody [20]. To optimize the duration of this treatment, we further enhanced the infectious dose to >1000 CFU of Mtb H37rv (high dose). Therefore, we initially examined the effects of a high-dose Mtb infection by analyzing the outcomes of infection in C57BL/6 and IL-17A^−/−^ mice (Figure 4).

According to our expectations, high-dose infection with Mtb led to significantly higher bacterial burdens in IL-17A^−/−^ mice when compared with wildtype animals by Day 29 post-infection (Figure 4A). The survival of C57BL/6 mice decreased to a median overall survival of 163 days, while IL-17A^−/−^ mice exhibited a dramatically reduced median overall survival of merely 27 days (Figure 4B). Remarkably, the histopathological analysis revealed the appearance of highly pronounced granulomatous lesions in parts of the IL-17A^−/−^ mice on Day 29 after high-dose Mtb infection (Figure 4C). Significant differences in the proportions of inflammatory cellular infiltration were, however, again not detected (Figure 4D).

**Figure 5:**
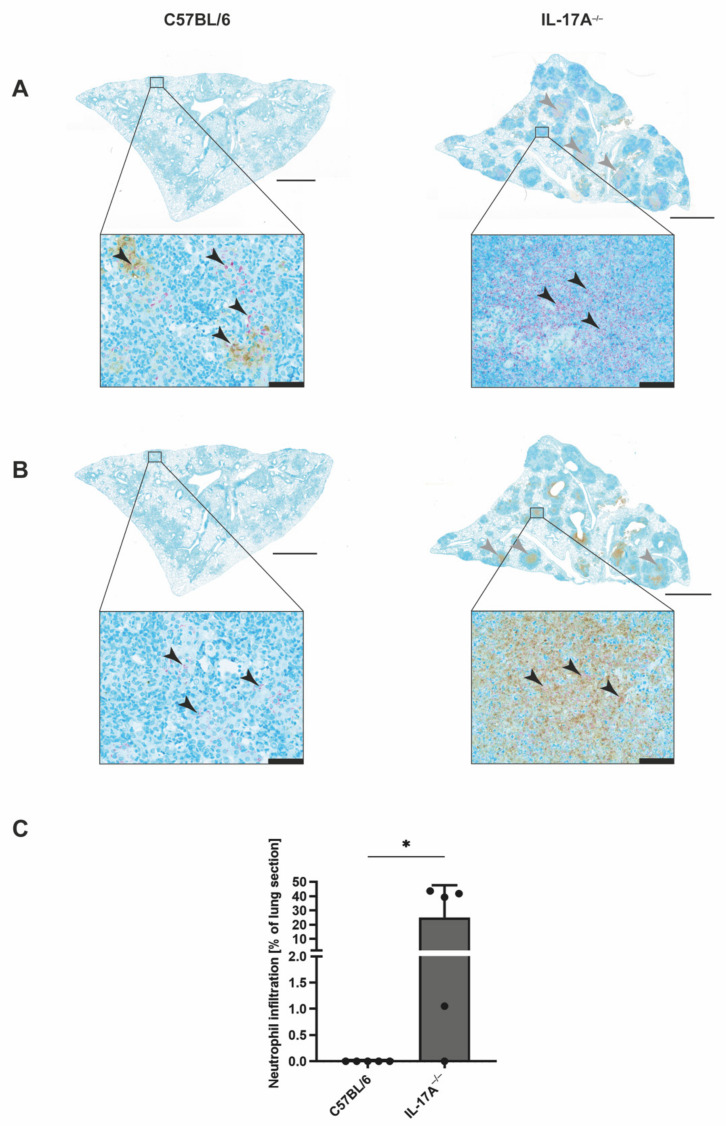
After ultrahigh-dose infection with Mtb, infectious lesions in the lungs of IL-17A^−/−^ mice are dominated by neutrophils containing high numbers of Mtb. (**A**–**C**) C57BL/6 and IL-17A^−/−^ mice were infected with >1000 CFU of H37rv Mtb by an aerosol. In formalin-fixed lung sections taken on Day 29 post-infection, acid-fast bacilli were detected by Ziehl–Neelsen staining. In addition, the sections were co-stained for NOS2 (**A**) or neutrophils (**B**,**C**). (**A**,**B**) Representative sections from 1 out of 5 mice per group are shown (larger images: magnification: 40×, bar: 2 mm; grey arrows: macrophage-containing lesions (**A**) or neutrophil-dominated lesions (**B**); detailed images: magnification: 400×, bar: 50 µm; black arrows: acid-fast bacilli). (**C**) Proportions of neutrophil infiltration within the total area of the lung sections were determined in 4–8 serial sections per animal. Data are presented as single dots and as the mean ± SD of 5 mice per group from one representative experiment. Statistics were determined using a Mann-Whitney test. Differences between C57BL/6 and IL-17A^−/−^ mice were defined as significant (* *p* < 0.05).

To investigate the presence of neutrophils in lung sections of IL-17A^−/−^ mice infected with high-dose Mtb H37rv, we again performed co-staining for acid-fast bacilli and NOS2 or neutrophils (Figure 5). Yet again, the staining exposed a pronounced lung infiltration of neutrophils in the absence of IL-17A (Figure 5B,C). While some of the clearly demarcated granulomatous lesions in the IL-17A^−/−^ mice contained Mtb-infected activated macrophages (Figure 5A), other lesions almost elusively comprised neutrophils in their center (Figure 5B). Moreover, neutrophils in these lesions exhibited high numbers of mycobacteria (Figure 5B). In contrast, the lungs of C57BL/6 wildtype mice showed diffuse granulomatous lesions that contain Mtb-infected activated macrophages (Figure 5A), whereas no considerable neutrophil infiltration was observable (Figure 5B). Together, these results clearly demonstrate that in the absence of IL-17A, the infiltration of neutrophils is exacerbated after infection with a high dose of Mtb H37rv. Moreover, these cells contained high amounts of mycobacteria, indicating that IL-17A deficiency results in unrestricted Mtb replication in the neutrophils.

**Figure 6:**
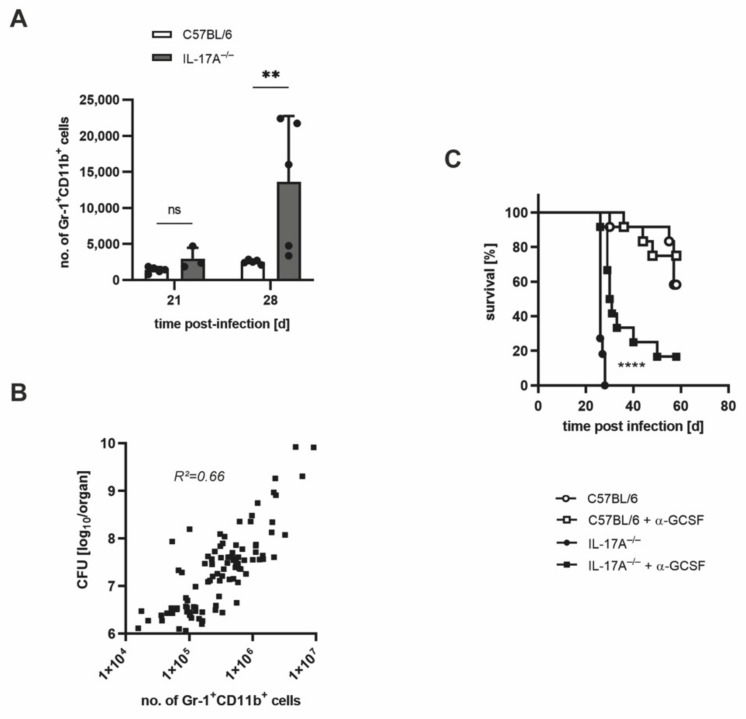
The increased neutrophil infiltration in the lungs of Mtb-infected IL-17A^−/−^ mice contributes to their increased susceptibility. (**A**) C57BL/6 and IL-17A^−/−^ mice were infected with >1000 CFU of H37rv Mtb by an aerosol. At different time points of infection, lung cells were phenotypically analyzed by flow cytometry. Absolute numbers of Gr-1^+^ CD11b^+^ neutrophils are shown. Data are presented as single dots and as the mean ± SD of 3–5 mice per group from one representative experiment out of two performed. Statistics were calculated using a two-way ANOVA with Bonferroni’s post hoc test. Differences between C57BL/6 and IL-17A^−/−^ mice were defined as significant (ns *p* ≥ 0.05; ** *p* < 0.01) (**B**) C57BL/6, IL-17A^−/−^, and IL-17A/F^−/−^ mice were infected with <100 or >1000 CFU of H37rv Mtb via the aerosol route. The correlation between bacterial loads and the absolute numbers of Gr-1^+^ CD11b^+^ neutrophils are shown, and the squared Pearson’s correlation coefficient was calculated (*n* = 95). (**C**) C57BL/6 and IL-17A^−/−^ mice were infected with >1000 CFU of H37rv Mtb via the aerosol route. To deplete neutrophils, 100 µL of an anti-G-CSF sheep antiserum was intravenously injected 5 and 3 days before infection with Mtb. The survival of 3–11 infected anti-G-CSF-treated and untreated mice of each strain was detected. Statistical analysis of the resulting survival curve was performed using the log-rank test. Differences in the survival kinetics between untreated and anti-G-CSF-treated IL-17A^−/−^ were defined as significant (**** *p* < 0.0001).

Quantitative flowcytometric analysis also revealed that after high-dose Mtb infection, the amount of CD11b^+^ Gr-1+ cells in the lungs of IL-17A^−/−^ mice was approximately five times higher than in the lungs of the corresponding wildtype mice on Day 28 post-infection (Figure 6A). In accordance with these findings, regression analysis of C57BL/6, IL-17A^−/−^, and IL-17A/F^−/−^ mice infected with different doses of Mtb H37rv revealed a significant positive correlation of the numbers of neutrophils and the bacterial loads in infected mice (Figure 6B). Finally, the depletion of neutrophils in C57BL/6 and IL-17A^−/−^ mice infected with >1000 Mtb H37rv revealed the significantly prolonged survival of IL-17A^−/−^ mice in the absence of neutrophils (Figure 6C). By determining the granulocyte numbers in blood smears, the efficiency of neutrophil depletion was proven beforehand (Figure S1).

Together, our results give evidence that after high-dose Mtb infection, the elevated neutrophil influx contributes to the increased susceptibility of IL-17A^−/−^ mice.

### 3.4. In IL-17A^−/−^ Mice, IL-17F Mediates Susceptibility to Elevated Infectious Doses of Mtb H37rv

**Figure 7:**
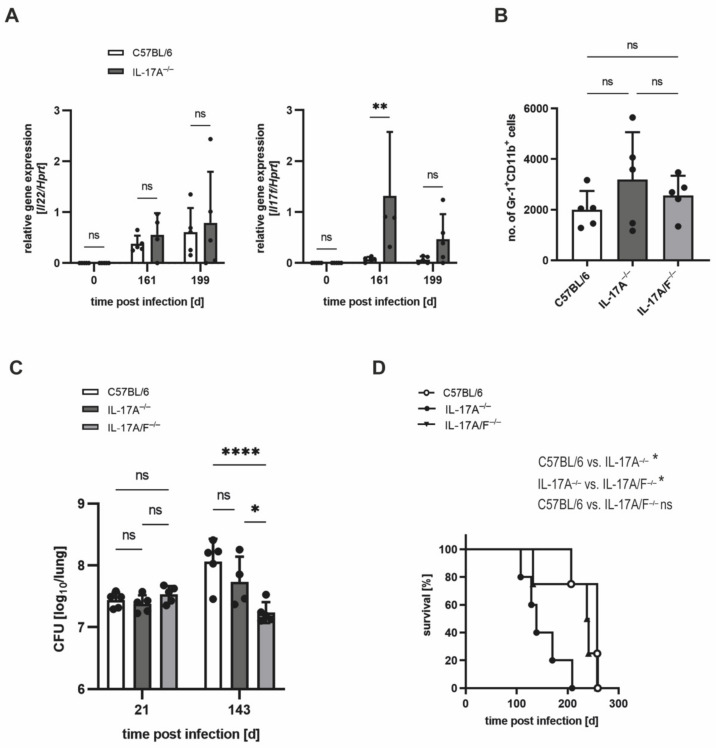
IL-17F mediates impaired survival in Mtb-infected IL-17A^−/−^ mice without exerting a significant influence on the numbers of neutrophils in the lung. (**A**–**D**) C57BL/6, IL-17A^−/−^, and IL-17A/F^−/−^ mice were infected with 100–1000 CFU of H37rv Mtb by an aerosol. (**A**) At different time points of infection, gene expression levels of *Il22* and *Il17f* were quantified by real-time RT-PCR in lung homogenates of C57BL/6 and IL-17A^−/−^ mice. Data are presented as single dots and as the mean ± SD of 4–5 mice per group from one representative experiment (*Il22*) or from one representative experiment out of two performed (*Il17f*). Statistics were calculated using a two-way ANOVA with Bonferroni’s post hoc test. Differences between C57BL/6 and IL-17A^−/−^ mice were defined as significant (ns *p* ≥ 0.05; ** *p* < 0.01). (**B**) On Day 21 post-infection, lung cells of C57BL/6, IL-17A^−/−^, and IL-17A/F^−/−^ mice were phenotypically analyzed by flow cytometry. Absolute numbers of Gr-1^+^ CD11b^+^ neutrophils are shown. Data are presented as single dots and as the mean ± SD of 5 mice per group from one representative experiment out of two performed. Statistics were calculated using Kruskal–Wallis’s test with Dunn’s post hoc test. Differences between C57BL/6, IL-17A^−/−^ and IL-17A/F^−/−^ mice were defined as significant (ns *p* ≥ 0.05). (**C**) At different time points of infection, CFUs in the lungs were determined. Data are presented as single dots and as the mean ± SD of 4–5 mice per group from one representative experiment. Statistics were calculated for log-transformed data using a two-way ANOVA with Bonferroni’s post hoc test. Differences between C57BL/6 and IL-17A^−/−^ mice, between IL-17A^−/−^ and IL-17A/F^−/−^ mice, or between C57BL/6 and IL-17A/F^−/−^ mice were defined as significant (ns *p* ≥ 0.05; * *p* < 0.05; **** *p* < 0.0001). (**D**) The survival of 4–5 infected C57BL/6, IL-17A^−/−^, and IL-17A/F^−/−^ mice was recorded using humane endpoints. Statistical analysis of the resulting survival curve was accomplished using the log-rank test. Differences in the survival kinetics between C57BL/6 and IL-17A^−/−^ mice, between IL-17A^−/−^ and IL-17A/F^−/−^ mice, or between C57BL/6 and IL-17A/F^−/−^ mice were defined as significant (ns *p* ≥ 0.05; * *p* < 0.05).

Our result that after infection with an elevated dose of Mtb, IL-17A^−/−^ mice partially exhibit enhanced recruitment of neutrophils in the lung is in stark contrast to the finding that IL-17A appears to be closely involved in the induction of neutrophils in response to infection with Mtb [15]. Because IL-17F has similar functions to IL-17A [24], we speculated that IL-17F may overcompensate for the absence of IL-17A, thereby mediating an overshooting neutrophil response. In fact, a gene expression analysis of lung homogenates from wildtype and IL-17A^−/−^ mice revealed that whereas *Il12p40*, *Il23p19*, *Il21*, and *Il22* were comparably induced after infection with an elevated dose of Mtb, the expression of the *Il17f* gene was significantly upregulated in the absence of IL-17A on Day 161 and tended to be higher on Day 199 post-infection (Figure 7A; Figure S2A–C). To determine whether IL-17F compensates for the absence of IL-17A and mediates the increased infiltration of neutrophils into the lungs of IL-17A^−/−^ mice, we infected IL-17A/F^−/−^ mice along with IL-17A^−/−^ and C57BL/6 mice with an elevated dose of Mtb H37rv (Figure 7B–D). Flowcytometric analysis of neutrophil infiltration into the lungs of infected animals, however, did not show a significant difference in the recruitment of Gr-1^+^ CD11b^+^ cells in IL-17A^−/−^ and IL-17A/F^−/−^ mice, although neutrophil numbers in IL-17A/F^−/−^ mice tended to be lower when compared with the IL-17A^−/−^ animals (Figure 7B). In contrast, determination of CFUs in the lungs revealed that on Day 143 after infection with an elevated dose of Mtb H37rv, IL-17A/F^−/−^ mice appeared to exhibit a significantly lower bacterial burden when compared with both the IL-17A^−/−^ mice and the wildtype controls (Figure 7C). When assessing these data, however, we must consider that, here, the analysis was performed prior to the onset of diminished mycobacterial containment and enhanced infiltration of neutrophils in the IL-17A^−/−^ mice. Importantly, we further observed that the increased susceptibility of IL-17A^−/−^ mice after infection with an elevated dose of Mtb was abolished by an additional deficiency of IL-17F, as the wildtype and IL-17A/F^−/−^ mice displayed similar survival times, whereas IL-17A^−/−^ mice had to be euthanized significantly earlier (Figure 7D). 

Together, our data give evidence that the compensatory expression of IL-17F in the absence of IL-17A mediates the increased susceptibility that was observed in the IL-17A^−/−^ mice infected with an elevated dose of Mtb. The infiltration of neutrophils in the absence of IL-17A, however, appears to be largely independent of the elevated expression of IL-17F in IL-17A^−/−^ mice.

## 4. Discussion

During experimental TB, the pro-inflammatory cytokine IL-17A appears to mediate protection against Mtb when produced at high levels. Thus, after infection with the Mtb isolate HN878, a pronounced induction of IL-17A correlates with an impact of the cytokine for early protective immune responses against the hypervirulent Mtb strain [7]. Similarly, IL-17A contributes to protection against Mtb H37rv in mice that lack the receptor for the Th17-suppressive cytokine IL-27 [6]. In these mice, IL-17A mediates the quality of protective T cell immune responses and the formation of highly stratified granulomas in the infected lungs. In contrast, in IL-17A^−/−^ mice, bacterial containment and protective immune responses to infection with ~ 100 CFU Mtb H37RV are not altered [7]. This latter finding was once more confirmed by the present study. Moreover, here, we demonstrated that, similar to what was observed in the context of hypervirulent Mtb, IL-17A contributes to protection after infection with an elevated dose of Mtb H37rv (probably mimicking higher virulence). In this context, IL-17A^−/−^ mice exhibit significantly higher bacterial burdens in the lungs, along with the significantly decreased survival of the mice. In accordance with the higher mycobacterial load, in the absence of IL-17A, infection with an elevated dose of Mtb H37rv results in the presence of larger inflammatory infiltrates in the lungs. The protective impact of IL-17A became even more clear after the infection of IL-17A^−/−^ mice with infectious doses of Mtb H37rv higher than 1000 CFU. Thereafter, the survival of IL-17A^−/−^ mice was massively diminished. Simultaneously, the lungs of high-dose Mtb-infected IL-17A^−/−^ mice showed extremely pronounced granulomatous lesions.

In the context of several mouse experimental models of both infection and autoimmunity, the expression of IL-17A has been connected to the migration of neutrophils [25,26,27]. During airway inflammation, the release of IL-17A also appears to trigger the accumulation of neutrophils in the lung [28]. After experimental infection with BCG, the absence of IL-17A results in a significant decrease in neutrophil recruitment to the infected lung, while mycobacterial containment is not affected [15]. On the other hand, the enhanced bacterial burdens in Mtb HN878-infected IL-17A^−/−^ mice were neither accompanied by a defect in the overall numbers of neutrophils in the lung, nor by an impaired accumulation of neutrophils within the lung granulomas [7].

Whereas these findings indicate that neutrophils may not function as direct effector cells against mycobacteria, studies with human neutrophils have suggested that these cells may be able to kill Mtb [29,30,31]. On the other hand, it was also shown that within human neutrophils, Mtb escapes from intracellular killing by ROS-mediated induction of necrosis [32]. Together, these studies create a conflicting picture of the potential of neutrophils to kill Mtb. Therefore, it may be likely that neutrophils function as a “Trojan horse”, hiding mycobacteria from activated macrophages [31]. Additionally, massive neutrophil infiltration may cause tissue damage and was, in the context of human TB, described as being involved in the transition to active disease [31,33]. Nonetheless, neutrophils also appear to be essential for the early formation of pulmonary granulomas in response to infection with Mtb via the induction of CXCR3-signalling chemokines [34]. 

The present study demonstrated that after high-dose infection with Mtb H37rv, the absence of IL-17A results in the accumulation of neutrophils within the centers of clearly pronounced inflammatory lesions. These neutrophils contain high numbers of mycobacteria, indicating largely unrestricted mycobacterial growth within these cells. Furthermore, a regression analysis revealed that the presence of Gr-1^+^ CD11b^+^ neutrophils in the lungs of Mtb H37rv-infected mice was inversely correlated with the degree of mycobacterial containment. Eventually, we were able to show that the survival of high-dose Mtb-infected IL-17A^−/−^ mice could be significantly enhanced by the depletion of neutrophils with an anti-G-CSF anti-serum. Consequently, the strong accumulation of Mtb-containing neutrophils in the absence of IL-17A indeed partially accounts for the increased susceptibility to infection with high doses of Mtb H37rv. Since we observed large numbers of mycobacteria within the cells, it is reasonable to assume that the neutrophils here serve as a survival niche that protects Mtb from the host’s effector mechanisms.

Our findings, in the first place, appear to contrast with earlier studies which demonstrated the close involvement of IL-17A in the migration of neutrophils in response to several infectious agents such as mycobacteria [15,25,26]. To find a possible explanation for the conflicting results, we examined whether other Th17-secreted cytokines may compensate for the absence of IL-17A by mediating the observed overshooting induction of neutrophils. While the expression of *Il21* and *Il22* was equal in the lungs of C57BL/6 and IL-17A^−/−^ mice infected with an elevated dose of Mtb H37rv, we found the closely IL-17A-related cytokine *Il7f* [24,35] to be significantly induced in the absence of IL-17A. By use of mice with a simultaneous deficiency in *Il17a* and *Il17f*, we were finally able to prove that the lack of IL-17F in IL-17A^−/−^ mice culminates in the complete recovery of the animals, as evident by the equal survival times of wildtype and IL-17A/F^−/−^ mice. However, the accumulation of neutrophils in the lungs does not appear to be further affected in the absence of both cytokines, indicating that IL-17F does not mediate the accrual of neutrophils in IL-17A^−/−^ mice infected with a high dose of Mtb. Together, our data demonstrate the compensatory potential of the Th17-secreted cytokines IL-17A and IL-17F in the context of experimental TB, as the highly elevated expression of IL-17F in the absence of IL-17A indeed mediated the increased susceptibility after infection with an elevated dose of Mtb H37rv. To further evaluate the actual impact of IL-17F and the underlying mechanism in the context of experimental TB, the outcome of a high-dose Mtb H37rv infection may be investigated in *Il7f* single-deficient mice. Likewise, analysis of Mtb HN878-infected IL-17F^−/−^ mice may be of interest.

Besides the massive influx of neutrophils, additional reasons for the enhanced susceptibility of IL-17A^−/−^ mice to high-dose infection with Mtb H37rv are conceivable. In this context, we analyzed the frequencies of IFN-γ-producing Th1 cells in the lungs of IL-17A^−/−^ mice infected with a moderately elevated or a high dose of Mtb H37rv (data not shown). The obtained results, however, did not indicate any quantitative impact of IL-17A on the pulmonary Th1 immune response against Mtb. In line with these data, IL-17A also does not affect the overall frequency of Th1 cells in the lungs of Mtb-infected IL-27Rα^−/−^ mice [6]. Instead, in these mice, the high expression of IL-17A contributes to the strategic positioning of IFN-γ, IL-2, and TNF co-expressing multifunctional CD4^+^ T cells in lung granulomas. To our knowledge, qualitative alterations in the protective Th1 immune responses have not yet been investigated in IL-17A^−/−^ mice infected with a high dose of Mtb H37rv. Future analyses may also reveal possible IL-17A-dependent changes in macrophage function.

## 5. Conclusions

In conclusion, our results surprisingly show that after experimental infection with high doses of Mtb H37rv, the absence of IL-17A provoked a strong accumulation of Mtb-containing neutrophils in the lung, which triggered susceptibility to Mtb. The enhanced susceptibility was furthermore mediated by the compensatory expression of IL-17F in the absence of IL-17A. Since the enhanced expression of IL-17F did not appear to mediate the infiltration of neutrophils into the lungs of IL-17A^−/−^ mice, this effect may only partially account for the attenuated susceptibility in the absence of IL-17A. The study therefore once more demonstrated how the delicate balance of cytokine-mediated immune responses against Mtb may affect the efficiency of mycobacterial containment. At the same time, timely restricted interference with cytokine-mediated protective immune responses may represent an advanced treatment option in the context of novel vaccination strategies or host-directed therapy approaches.

## Data Availability

The datasets generated during and/or analyzed during the current study are stored on an internal server at the Research Center, Borstel, and are available from the corresponding author on reasonable request.

## Supplementary Materials

The following supporting information can be downloaded at: https://www.mdpi.com/article/10.3390/cells11182875/s1, Figure S1: In vivo injection of C57BL/6 and IL-17A^−/−^ mice with anti- GCSF results in the efficient depletion of neutrophils after very-high-dose infection with Mtb; Figure S2: After infection with an elevated dose of Mtb, expression of the genes Il12p40, Il23p19, and Il21 in lung homogenates from C57BL/6 and IL-17A^−/−^ mice was comparably.
