# Supplementary material for: High-Dose Mycobacterium tuberculosis H37rv Infection in IL-17A- and IL-17A/F-Deficient Mice
Source: Cells. 2022 Sep 14;11(18):2875. doi: 10.3390/cells11182875 (PMC9496946; doi:10.3390/cells11182875)
Supplement: Supplementary file 1 [file cells-11-02875-s001.zip › cells-1774757-supplementary.pdf]

High dose Mycobacterium tuberculosis H37rv infection in IL-17A- and IL-17A/F-deficient mice

Supplementary materials

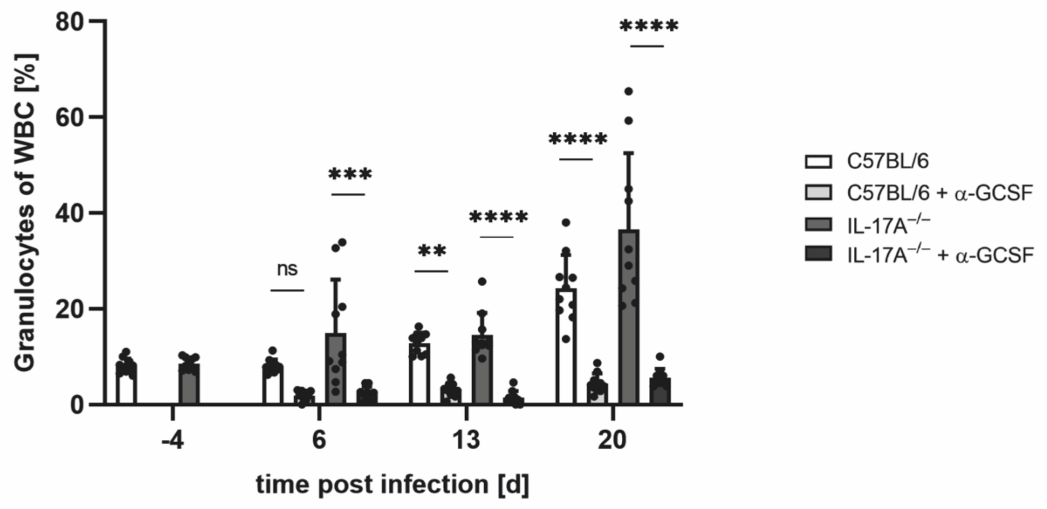

Figure S1. In vivo injection of C57BL/6 and IL-17A$^{-/-}$ mice with anti- GCSF results in the efficient depletion of neutrophils after very-high-dose infection with Mtb. C57BL/6 and IL-17A$^{-/-}$ mice were infected with > 1000 CFU of H37rv Mtb by an aerosol. To deplete neutrophils, 100 µl of an anti-G-CSF sheep antiserum was intravenously injected 5 and 3 days before infection with Mtb. At different time points after infection with Mtb, the proportion of granulocytes within the total amount of white blood cells (WBC) was determined in blood smears from 5 mice per group from one experiment. Data are presented as single dots and as the mean ± SD mean ± SD. Statistics were conducted for days 6, 13 and 20 after infection using a two-way ANOVA with Bonferroni post hoc test. Differences between treated and non-treated C57BL/6 and between treated and non-treated IL-17A$^{-/-}$ mice were defined as significant (ns $p \geq 0.05$; **$p < 0.01$; ***$p < 0.001$; ****$p < 0.0001$).

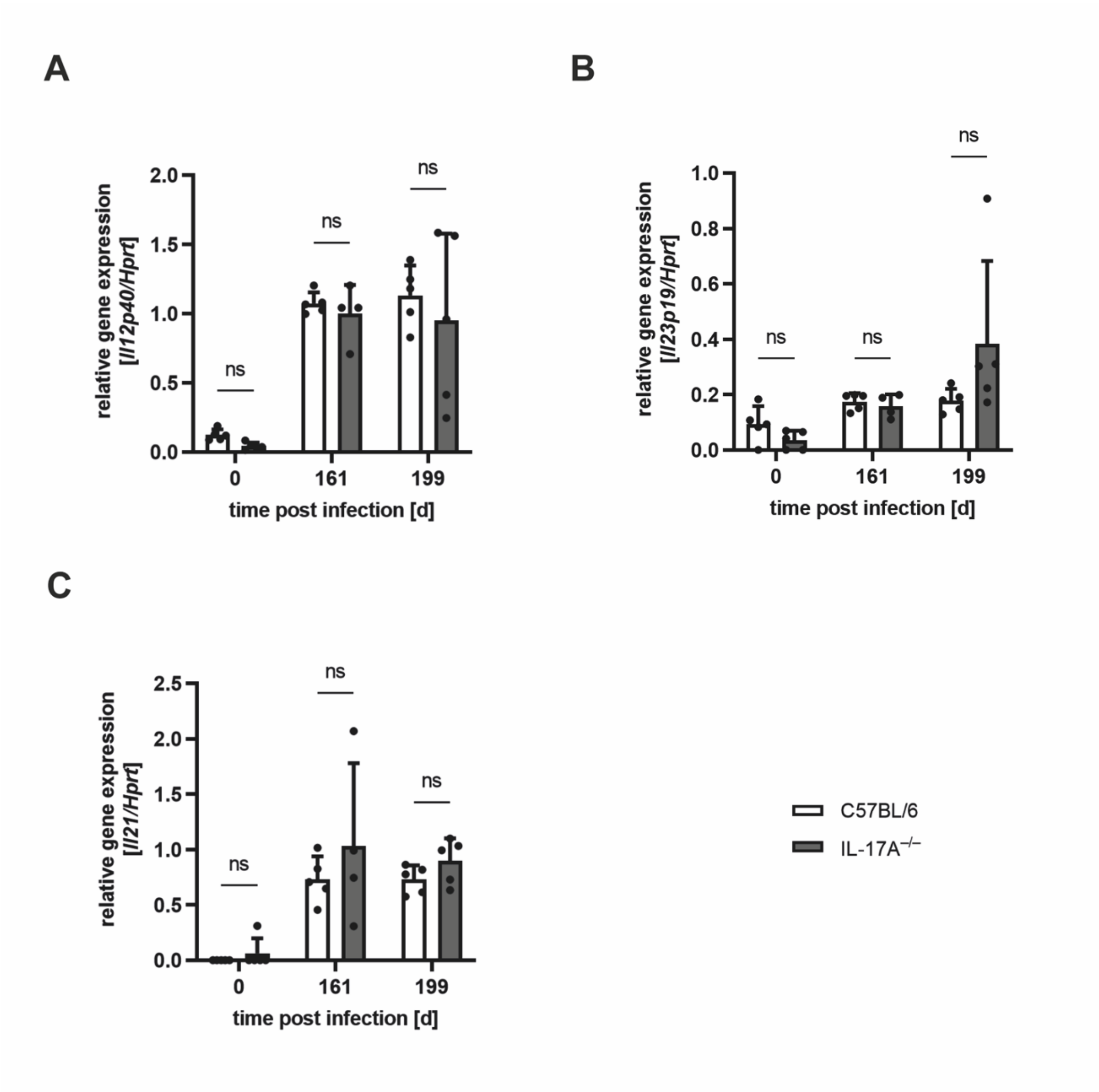

Figure S2. After infection with an elevated dose of Mtb, expression of the genes *Il12p40*, *Il23p19*, and *Il21* in lung homogenates from C57BL/6 and IL-17A$^{-/-}$ mice was comparably. (A-C) C57BL/6 and IL-17A$^{-/-}$ mice were infected with 100-1000 CFU of H37rv Mtb by an aerosol. (A) At different time points of infection, gene expression of *Il12p40* (A), *Il23p19* (B) and *Il21* (C) were quantified by real-time RT-PCR in lung homogenates. Data are presented as single dots and as the mean ± SD mean ± SD of 4-5 mice per group from one representative experiment. Statistics were conducted using a two-way ANOVA with Bonferroni post hoc test. Differences between C57BL/6 and IL-17A$^{-/-}$ mice were defined as significant (ns $p \geq 0.05$).
